# Structural Insights into Influence of Isomerism on Properties of Open Shell Cobalt Coordination System

**DOI:** 10.3390/molecules24183357

**Published:** 2019-09-16

**Authors:** Marcin Swiatkowski, Tomasz Sieranski, Marta Bogdan, Rafal Kruszynski

**Affiliations:** Institute of General and Ecological Chemistry, Lodz University of Technology, Zeromskiego 116, 90-924 Lodz, Poland; marcin.swiatkowski@p.lodz.pl (M.S.); tomasz.sieranski@p.lodz.pl (T.S.); 801084@edu.p.lodz.pl (M.B.)

**Keywords:** cobalt, butyrate, isobutyrate, aminopyridine, oxide, nanoparticles, TD-DFT, IR, UV-Vis

## Abstract

The two coordination compounds of cobalt were designed and synthesized. The substrates were carefully selected to allow gentle tuning of the molecular structure of the designed compounds. The crystal, molecular and supramolecular structure of studied compounds has been determined and discussed. The spectroscopic and thermal properties of designed coordination compounds have been studied and their application as precursors for the synthesis of cobalt oxide nanoparticles has been demonstrated. It was proven that not only are parameters of conversion of the precursor to nanoparticles important, but also small changes in molecular structure can considerably affect the size of formed particles. For unambiguous determination of the influence of compounds structure on their UV-Vis radiation absorption, density functional theory and time-dependent density functions theory calculations have been performed. The complexity of the correct ab-initio reflection of the open shell molecular system was outlined and discussed. The results obtained from density functional theory (DFT) calculations have been also employed for discussion of the bonding properties.

## 1. Introduction

Recently, extensive attention has been focused on the coordination chemistry of transition metals carboxylates, especially with N-donor ligands [1,2,3,4]. Coordination compounds belonging to this group are especially interesting because of their useful properties and wide applications (e.g., in wood preservation, as photoluminescent materials, in thermoelectric devices and magnetic materials) originating from their specific crystal structures [1,5,6]. Due to possessing various coordination modes, the carboxylate anions have been used for the synthesis of compounds with diverse structures and topologies. These anions exhibit excellent functional properties in terms of their strong coordination abilities. Because of their versatility, at the specific conditions they can adopt one of the wide of bonding modes (e.g., monodentate, bidentate, tridentate, symmetric, asymmetric, bridging, chelating) [7,8]. The introduction of the properly selected other ligand (in the studied case an N-donor one), leads to the increase in the structural diversity and improves the functionality of a compound. Additionally, such a ligand affects the binding mode of carboxylate anions due to competition in both the inner and outer coordination sphere. For these purposes, good candidates are ligands derived from pyridine, mainly due to the presence of an exposed heterocyclic nitrogen atom able to coordinate easily to the metal ions. Such N-donor ligands found tremendous applications in many fields, e.g., in pharmacy and medicine, as a result of their biological activity and ways in which they can bond to metal ions [9]. The strength of the nitrogen coordination site of a pyridine ring originates from a possibility of overlapping of the sp^2^ nitrogen lone pair orbital with hybridized metal orbitals [10,11]. This interaction can cause a large splitting of metal d-orbitals influencing compound spectral and magnetic properties [12]. Apart from the interactions involving its nitrogen lone pair orbitals, the pyridine ring can be engaged in other interactions such as hydrogen bonding, π•••π stacking and cation •••π interactions. In case of such N-donor ligands, the substituents at position 2 and/or 6 of the ring play a significant role in its interactions. For example, the amino groups may stabilize the compound structure with hydrogen bonds and can donate electrons disfavoring π•••π stacking interactions [13,14]. The simplest ligand belonging to this subgroup, 2-aminopyridine (2-apy), can form coordination compounds with almost all transition metal ions. It usually acts as a monodentate ligand coordinating through its pyridine nitrogen atom. In specific cases it can also serve as a bidentate ligand, involving both N atoms (pyridine and amine) in coordination to metal centers [15].

Among transition elements, cobalt is one of the most important due to its versatile and valuable properties (among others dyeing, magnetic and catalytic) [16]. The variety of cobalt coordination compounds are known, however, only 5 compounds containing carboxylate anions and 2-apy directly coordinated to the metal cation were structurally determined [17]. These carboxylate anions were as follows: valproate [18], benzoate [19,20], pivalate [21], oxalate [22] and acetate [1]. All these compounds are mononuclear apart from one containing oxalate anions in which 1D zig-zag coordination polymer was formed (oxalate anions act as bridging ligands). As it has been shown, the coordination chemistry of cobalt carboxylates with 2-apy is poorly explored. Hence, a combination of carboxylate anions (butyrate and isobutyrate) and an N-donor heterocyclic ligand (2-apy) has been utilized for the synthesis of studied cobalt coordination compounds. The appliance of butyrate and isobutyrate allowed the determination of the influence of the isomerism within carboxylate aliphatic chain on the structure and spectral and thermal properties of the synthesized compounds. It is especially important in crystal engineering, where small adjustments of properties are frequently required, and such adjustments can be attained by proper selection of isomers of compounds possessing the same empirical formula.

The spectroscopic and thermal properties of designed coordination compounds have been studied and their application as precursors for the synthesis of cobalt oxide nanoparticles has been proven. Generally, in the single precursor method, the parameters of conversion of precursor to nanoparticles are studied, however, it was demonstrated that size and morphology of nanoparticles strongly depend on the structure of a coordination precursor [23,24]. Cobalt(II) oxide nanoparticles have numerous applications e.g., in lithium-ion batteries [25,26], gas sensors [27,28], as catalysts in Fischer-Tropsch synthesis, in oxygen evolution reaction or photodegradation of diazo dyes [29,30,31] and as a pigment in ceramic or rubber industry [32,33]. For unambiguous determination of the influence of compounds structure on their UV-Vis radiation absorption, density functional theory (DFT) and time-dependent density functions theory (TD-DFT) calculations have been performed. DFT and TD-DFT are commonly used to study the electronic structure of coordination compounds of transition metals [34,35]. They are easy to use and fast enough to study even relatively large molecules [36]. The results obtained from DFT calculations have also been employed for discussion of the bonding properties.

## 2. Materials and Methods

### 2.1. Synthesis

All reagents were bought from Sigma-Aldrich (St. Louis, MO, USA) and were analytical grade. The analytical grade water was prepared from improved drinking water via combined reverse osmosis and ion exchange processes.

#### 2.1.1. Synthesis of Cobalt Carboxylates

The respective cobalt carboxylate (i.e., cobalt butyrate and cobalt isobutyrate) was synthesized by suspending the cobalt oxide (3 mmol, 0.2248 g) in a standard solution of the appropriate carboxylic acid (5 mmol of acid in 50 cm^3^ of water, c = 0.1 mol∙dm^−3^). These mixtures were heated at 100 °C under a reflux condenser for 3 h. Then, they were filtered in order to remove an unreacted excess of cobalt oxide. The concentrations of the respective cobalt salts (butyrate and isobutyrate) in the final solutions were determined via edta titration of cobalt cations (at pH = 9.25, in presence of pyrogallol red as an analytical indicator) [37], and they were 0.0472 and 0.0458 mol∙dm^−3^ respectively, for cobalt butyrate and cobalt isobutyrate.

#### 2.1.2. Synthesis of Coordination Compounds

The coordination compounds were synthesized via a direct reaction between the respective cobalt carboxylate and 2-aminopyridine (2-apy). Two syntheses with different molar ratio of substrates (i.e., 1:1 and 1:2) were carried out for each pair of the respective cobalt carboxylate and 2-apy. The solutions containing 1 mmol of the appropriate cobalt carboxylate (21.2 cm^3^ and 21.8 cm^3^ of the above described solutions, respectively) were mixed with the appropriate volumes of water solution containing 1 and 2 mmol of 2-apy, respectively (the concentration of the used 2-apy solution was 0.5 mmol∙cm^−3^). The reaction mixtures were stirred vigorously at room temperature for 30 min and they were left to crystallize at 4 °C. Violet crystals of (**1**) [Co(*n*-C_3_H_7_COO)_2_(2-apy)_2_] and (**2**) [Co(*i*-C_3_H_7_COO)_2_(2-apy)_2_] grew after 4–5 weeks. Elemental analyses (calculated/found (%)) for **1**: C 51.31/50.98; H 6.22/6.31; Co 13.99/14.14; N 13.30/13.24; O 15.19/15.37 and for **2**: C 51.31/51.18; H 6.22/6.15; Co 13.99/14.28; N 13.30/13.11; O 15.19/15.31.

#### 2.1.3. Synthesis of Cobalt(ii) Oxide Nanoparticles

Nanoparticles of cobalt oxide were synthesized using a single precursor method based on the thermal conversion of the synthesized coordination compounds (precursors) in a muffle furnace. Two variants of this method were applied. In the first one, samples of precursors (50 mg each) were heated with a heating rate of 5 °C∙min^−1^, up to the temperature of CoO formation (370 °C, based on the thermal analyses of the coordination compounds), in air atmosphere [38]. In the second variant, precursors samples (50 mg each) were suspended in oleic acid (1.0 cm^3^) and heated at 220 °C for 2 h. After this process, a small volume of toluene (0.5 cm^3^) and a large excess of methanol (10 cm^3^) were added to the suspensions, and black precipitates were separated by centrifugation. They were washed three times with 5 cm^3^ of methanol and dried in air [39,40]. The identity of the bulk phase was confirmed by X-ray powder diffraction (as described below) and the purity from organic molecules and fragments was determined with high resolution IR spectrometry (all spectra did not contain bands of any organic oscillators).

### 2.2. Crystal Structure Determination

X-ray intensity data of compound **1** were collected on the Kuma KM-4-CCD automatic diffractometer (Kuma Diffraction, Wroclaw, Poland) equipped with the CCD detector (Kuma Diffraction, Oxford, UK) and CuKα1 radiation (λ = 1.54178 Å) at the temperature of 100.0(1) K, with ω scan mode. X-ray intensity data of compound **2** were collected on the Synergy Dualflex Pilatus 200K automatic diffractometer (Rigaku Corporation, Tokyo, Japan) equipped with the CCD detector and CuKα1 radiation (λ = 1.54184 Å) at temperature of 100.0(1) K, with ω scan mode. The structures were solved by the dual-space algorithm. All non-hydrogen atoms were refined anisotropically using the full-matrix, least-squares method on F2. All hydrogen atoms were found from difference Fourier syntheses and were refined in the riding model. Isotropic displacement factors of hydrogen atoms were equal to 1.2 times the value of an equivalent displacement factor of patent primary nitrogen atoms, secondary and tertiary carbon atoms and 1.5 times of patent primary carbon atoms. Carbon-bonded hydrogen atom positions were idealized after each cycle of refinement. The methyl groups were allowed to rotate about their local three-fold axes. The SHELXT [41], SHELXL [42], and SHELXTL [43] programs were used for all calculations. The selected details concerning crystals data and refinement of the studied coordination compounds are given in Table 1. CCDC 1,945,914 and 1,945,915 contains the supplementary crystallographic data for this paper. These data can be obtained free of charge via http://www.ccdc.cam.ac.uk/conts/retrieving.html (or from the CCDC, 12 Union Road, Cambridge CB2 1EZ, UK; Fax: +44 1223 336033; E-mail: deposit@ccdc.cam.ac.uk)

### 2.3. Other Physical Measurements

The IR spectra of the coordination compounds were recorded on the Jasco FT/IR 6200 spectrophotometer (JASCO, Easton, MD, USA), in the spectral range 4000–400 cm^−1^, in a form of KBr pellets. The UV-Vis diffuse reflectance spectra were recorded on the Jasco V-660 spectrophotometer (JASCO, Easton, MD, USA), in the spectral range 190–800 nm, using BaSO_4_ as a standard with 100% reflectance. The thermal analyses were carried out in the TG/DTA-SETSYS-16/18 thermoanalyser (SETARAM Instrumentation, Caluire, France) coupled with the ThermoStar (Balzers) mass spectrometer. Samples were heated in platinum crucibles up to 1000 °C, with heating rate 5 °C∙min^−1^ in synthetic air (21.0% O_2_, 79.0% N_2_) flow. Final products of decomposition were confirmed by X-ray powder diffraction (XRPD) using the Powder Diffraction File [44]. The X-ray powder diffraction (XRPD) patterns were measured in reflection mode on the XPert PRO XRPD system (Malvern Panalytical Ltd., Royston, UK) equipped with CuKα1 radiation, Bragg–Brentano PW 3050/65 high-resolution goniometer and PW 3011/20 proportional point detector. Surface morphologies of cobalt(II) oxide nanoparticles were characterized by the scanning electron microscope with energy dispersive X-ray spectrometer (SEM-EDS, HITACHI S-4700, EDS Thermo NORAN).

### 2.4. Quantum-Mechanical Calculations

The excited states of the studied coordination compounds have been calculated for X-ray-determined coordinates using TD-DFT. All the calculations were performed using Gaussian09 rev. D.01 [45] with B3LYP/6-31g++(2d,2p) level of theory. Input structural models were prepared with Mercury CSD 3.10.2 [46]. Positions of the hydrogen atoms have been normalized by moving them along the covalent bond vector (X→H) to the X–H distance equal to the average neutron diffraction value. One coordination unit was used as an input for one calculation. Compound **1** possesses two coordination units in the asymmetric part of the unit cell. Because their geometries differ slightly, the calculations have been performed for both of them. The differences in their calculated spectra were negligible (their spectra differ in terms of the oscillator strengths which are assigned to the particular excitation states, Figure S1 in the Supporting Materials), thus in further analysis the data of one calculation was used. The quartet ground state was an input as this state was more energetically favorable. The studied coordination compounds are relatively large in size, thus the number of calculated transitions was set to 130 for each compound, to include all the experimentally-founded absorption bands. The assignment of the calculated excited states to the observed experimental maxima was based on both the comparison of their excitation energies and the oscillator strengths/intensities of the corresponding maxima. The analysis of the character of respective orbital excitations was based on their contour plots.

## 3. Results and Discussion

### 3.1. Structural Analysis

**Figure 1:**
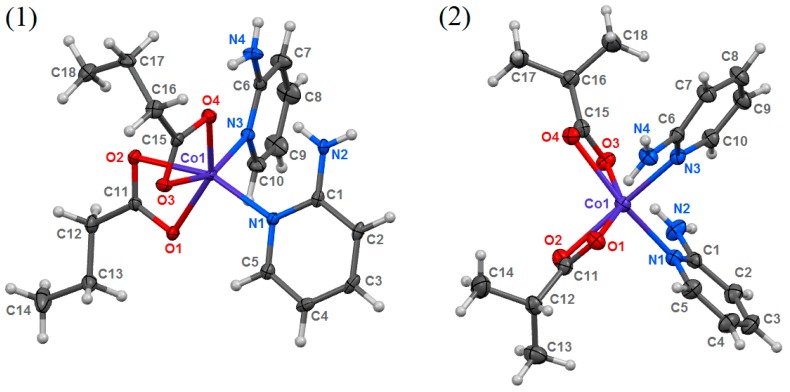
Solid state structures of the studied compounds (**1** and **2**), with atom numbering scheme, plotted with 50% probability of displacement ellipsoids of non-hydrogen atoms. Hydrogen atoms are plotted as spheres of arbitrary radii. For **1**, one of two isomorphous coordination entities from the asymmetric unit is presented.

**Figure 2:**
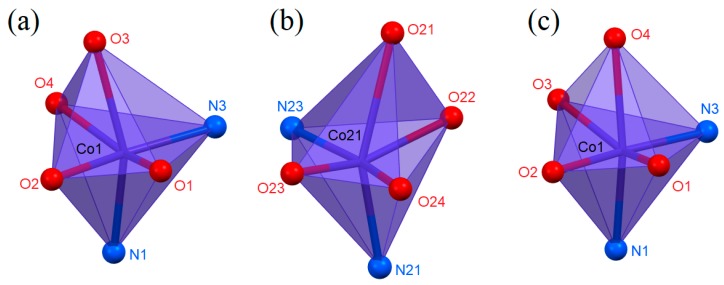
Coordination polyhedra of compound **1** (**a**,**b**) and **2** (**c**).

Reactions between cobalt(II) carboxylates and 2-aminopyridine (2-apy) led to the formation of two new coordination compounds, namely (**1**) bis(2-aminopyridine-κ*N*)bis(butyrate-κ^2^*O,O*′)cobalt(II) and (**2**) bis(2-aminopyridine-κ*N*)bis(isobutyrate-κ^2^*O,O*′)cobalt(II). These compounds were formed regardless of the substrates’ stoichiometry used in reactions. The metal-to-ligand ratio in them is 1:2, as a consequence of higher stability constant of Co(2-apy)_2_^2+^ system than Co(2-apy)^2+^ system (1.96 versus 1.78 [47]). The coordination entities in both compounds are similar (Figure 1). Cobalt cations are bonded with two monodentate 2-apy molecules coordinating by the nitrogen atom of the aromatic ring and two bidentate chelating carboxylate anions. Coordination unit containing Co21 cation (compound **1**) contains one bond (Co21—O21) noticeably longer than others (Table 2). To ensure that this is a real coordination bond, the total electron density distribution was analyzed. This analysis confirmed the existence of Co21-O21 bond (Figures S2 and S3 in the Supporting Materials). Additionally, the measured electron density (F_o_ generated from the observed diffraction intensities) in the Co21/O21/O22 plane of compound **1** (Figure S4 in the Supporting Materials) also show the positive values along linkage of Co21—O21 and Co21—O22, as well as the negative values along the line segment Co21–C31, what also proves the formation of the Co21–O21 coordination bond (there are two different electron density paths linking the cation with the carboxylate anion). Hence, the coordination number of all central atoms is 6. Coordination polyhedra adopt distorted octahedral geometries with *cis* stereolocation of ligands (Figure 2) [48]. The asymmetric unit of **1** contains two structurally independent coordination entities. They vary slightly in deformation of a coordination sphere. The sum of deviations of the 12 smallest inner angles of coordination polyhedra from 90° is 131.21° and 140.33° respectively, for Co1 and Co21 entities of **1** (Table 2). Distortions from ideal octahedral geometry observed in both compounds are caused by the rigid and flat structure of 2-apy and the bulky shape of carboxylate anions. Strong tensions arising around central atoms disallow occupation of ideal positions by coordinating atoms of ligands. 

**Figure 3:**
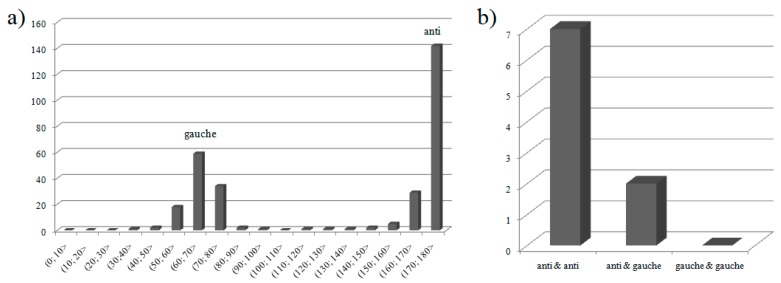
(**a**) Distribution of torsion angles between carbon atoms in coordinating butyrate anions (on the basis of Cambridge Structural Database, overall: 298 butyrate anions from 89 compounds) and (**b**) population of mononuclear coordination entities containing two metal-coordinating butyrate anions with definite conformations (overall 9 coordination entities from 5 compounds).

Butyrate anions in each entity of **1** exist in two conformations, anti and gauche, which is not typical phenomenon. The anti-conformation is only slightly more preferred among coordinating butyrate anions in all structurally characterized compounds (Figure 3a) [17], however considering only mononuclear compounds similar to the studied ones (i.e., containing two coordinating butyrate anions in inner coordination sphere), the entities with two anti butyrate anions dominate (Figure 3b).

#### 3.1.1. The Bond Valences

The bond valences were computed as *ν*_ij_ = exp((*R_ij_*-*d_ij_*)/*b*) [49,50], where *R_ij_* is the bond-valence parameter (in the formal sense *R_ij_* can be considered as a parameter equals to the idealized single-bond length between *i* and *j* atoms for the given *b* [51,52]) and *b* was taken as 0.37 Å [53,54]. The *R*_Co-N_ and *R*_Co-O_ were 1.727 [38] and 1.685 [55], respectively. The computed bond valences are included in Table 2. The computed total valences of central atoms (BVS) are 1.830 and 1.831 for 1 and 1.851 for 2, so they are close to the expected value of +2 (formal oxidation state of metal). In each case, a deviation of BVS is smaller than the value of 0.25–0.30 v.u. [56], which if exceeding, could indicate mistakes in an interpretation of the compound structure or in a charge of an ion. The BVS smaller than formal oxidation state of metal proves that the longest Co21-O21 distance represents real coordination bond. The observed deviations originate from tensions in the inner coordination spheres of cobalt cations, caused by the rigid and flat structure of 2-apy and relatively bulk chelating carboxylate anions. The Co—N bond strengths are similar in all compounds (falls in the range 0.35–0.39 v.u.) and are close to the strength of the one Co—O bond formed by each carboxylate ion (0.34–0.41 v.u.). In each coordination unit, the second Co—O bond formed by each carboxylate ion is distinctly weaker (0.08–0.24 v.u.). The asymmetricity of bonding of carboxylate ions is the largest in the coordination unit containing Co21 cation (the difference between the sum of the stronger and weaker Co—O bonds is 0.51 v.u.) and smaller for coordination unit containing Co1 cations in both compounds (these values are equal to 0.29 and 0.26 v.u. respectively, for compounds **1** and **2**). 

#### 3.1.2. Intermolecular Interactions

In both compounds, the sole donors of classical hydrogen bonds are amino groups and they form only N—H•••O interactions with carboxylate anions (Table 3). Each hydrogen atom of each amino group is involved in one H-bond. Moreover, each amino group forms one intramolecular and one intermolecular H-bond. In both compounds, chains motifs of unitary graph set assemble coordination entities along the crystallographic [100] axis. Two finite D motifs of unitary graph set of 1 form together C_2_^2^(14) pattern of the second level graph and this motif expands along the (010) axis. The remaining patterns of the binary graph set of 1 are diverse finite D motifs. The binary graph set of 2 consists of chain patterns C_2_^2^(14) and C_4_^4^(24) (extending along the (001) and (101) axes, respectively) and diverse R_6_^6^(36) patterns formed by the combination of first level ring and chain motifs. The crystal structure of 2 is also stabilized by π•••π stacking interactions [57]. An association exists between rings containing N3 and N21 atoms and it connects coordination entities in dimers. The associated rings are rotated to each other at about 177°, a distance between ring centroids is 4.1704(2) Å, perpendicular distances between centroid of one ring (containing N3 atom) and plane of second ring (containing N21 atom) is 3.6324(2) Å, a dihedral angle between ring planes is 8° and an angle between vector Cg(N3)→Cg(N21) and normal to plane containing N3 atom is 35°.

### 3.2. UV-Vis Spectrometric Analysis

**Figure 4:**
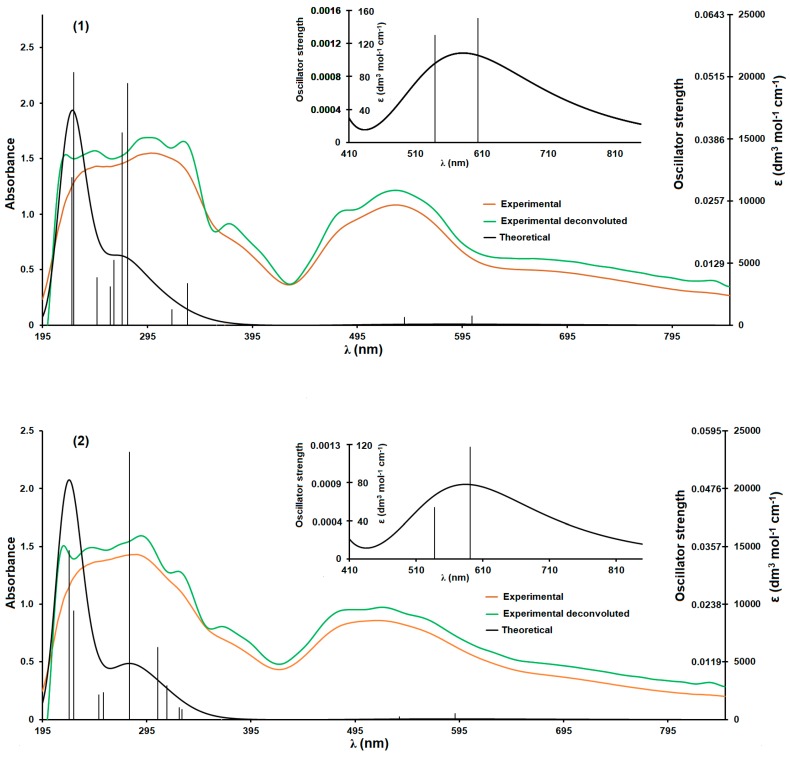
Experimental and calculated UV-Vis spectra of the studied compounds (**1** and **2**). The most important oscillator strengths are shown as vertical black lines.

**Figure 5:**
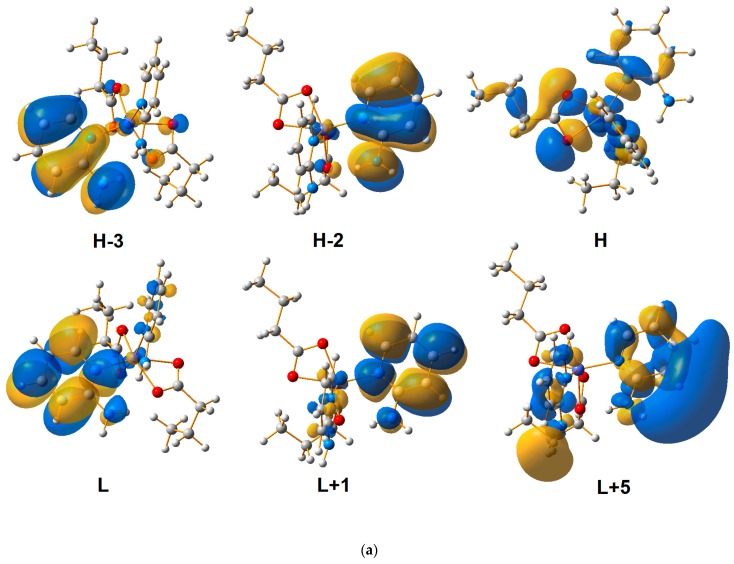

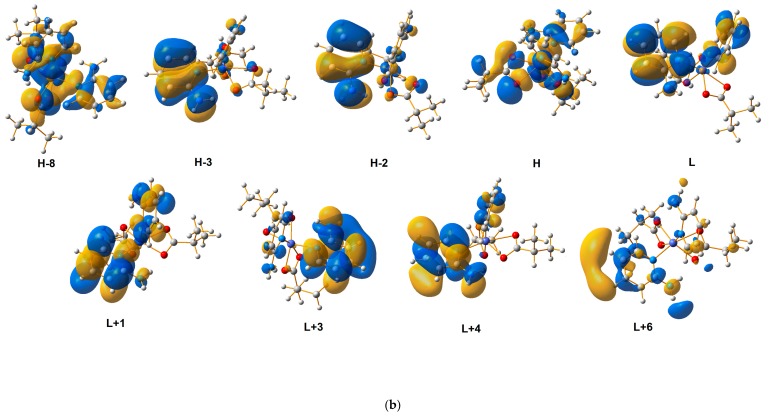

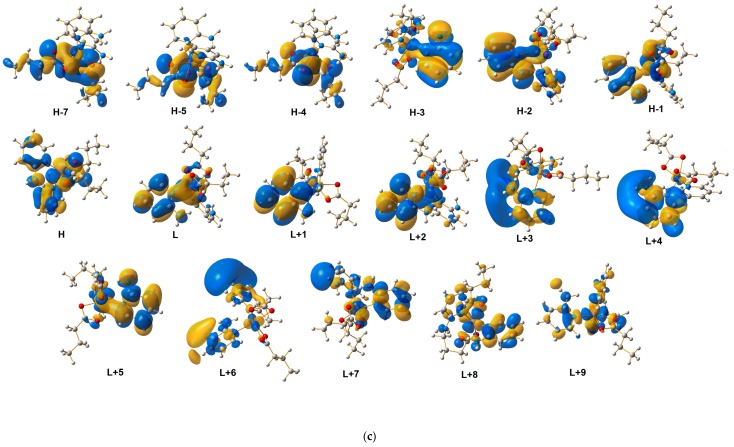

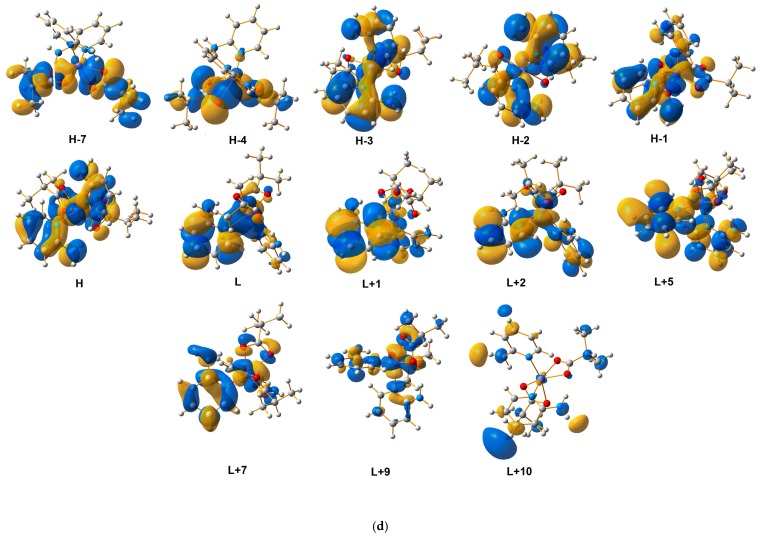
Calculated α molecular orbitals (**a**) of compound **1**, (**b**) of compound **2**, and β molecular orbitals (**c**) of compound **1**, (**d**) of compound **2**.

The solid-state spectra of the investigated compounds exhibit six maxima (Figure 4, Table 4). Generally, in the case of compound **1**, the maxima are observed at slightly higher wavelength numbers compared to compound **2**. An exception is the maximum observed at about 490 nm (Table 4). The calculated spectra (Figure 4, Table 4) are comparable to experimental ones. The exceptions are the bands observed at the longest wavelengths of the experimental spectra (ranging from 410 to 850 nm, Figure 4). In calculated spectra (Figure 4) these maxima are much less intense since their calculated oscillator strength values are relatively small (Table 4). It must be outlined that for the TD-DFT method, the accurate reproducing of UV-Vis spectra of Co(II) coordination compounds is difficult. The TD-DFT method works better for closed-shell systems [58]. For open-shell systems, the calculated spectra are slightly less accurate, but the results are still reliable since the excited states themselves are correctly reproduced (only the population of transitions is smaller than measured). Both studied compounds contain open-shell systems and thus each coordination compound possesses two distinguishable sets of orbitals (α and β ones, Figure 5). Generally, the transitions involving β orbitals (Figure 5c,d) dominate in studied coordination moieties (Table 4). The transitions are relatively complex and involve multiple different orbitals. The first observed absorption maximum is associated with π→π* transitions (Table 4, Figure 5a,b), however, especially for **2**, this maximum is also associated with ligand-to-metal charge transfer (LMCT) and ligand-to-ligand charge transfer (LLCT) transitions (the non-bonding orbitals of isobutyrate anions are involved in these two transitions; Table 4, Figure 5d). It suggests a larger contribution of this interaction to coordination bond in **2** than in **1**. Second maximum is mainly associated with n→π* transitions. This band is observed in the solid-state spectrum of pure 2-apy but in the spectra of the coordination compounds it is red shifted (Table 4). This effect is associated with differences in the dominant transition producing respective maxima. In the spectrum of 2-apy this band is attributed to π→π* transitions, [59] while in studied compounds n→π* transitions are observed and they are related to LLCT, which, apart from molecular orbitals of 2-apy, involve the non-bonding orbitals of butyrate and isobutyrate anions. The π→π* and n→π* transitions produce the third observed maximum (Table 4, Figure 5). They predominantly engage molecular orbitals of 2-apy (analogous maximum also exists in the solid-state spectrum of the pure ligand) [59]. Similar to the second maximum, the fourth maximum originates mostly from n→π* LLCT transitions involving non-bonding orbitals of butyrate and isobutyrate anions. Next, maximum, to a large extent, is associated with typical π→π* transitions (Table 4). The last two maxima are produced by complex orbital transitions involving d orbitals of cobalt cations, non-bonding orbitals of butyrate and isobutyrate anions and non-bonding and π orbitals of 2-apy ligand (Table 4, Figure 5c,d).

### 3.3. IR Spectrometric Analysis

**Figure 6:**
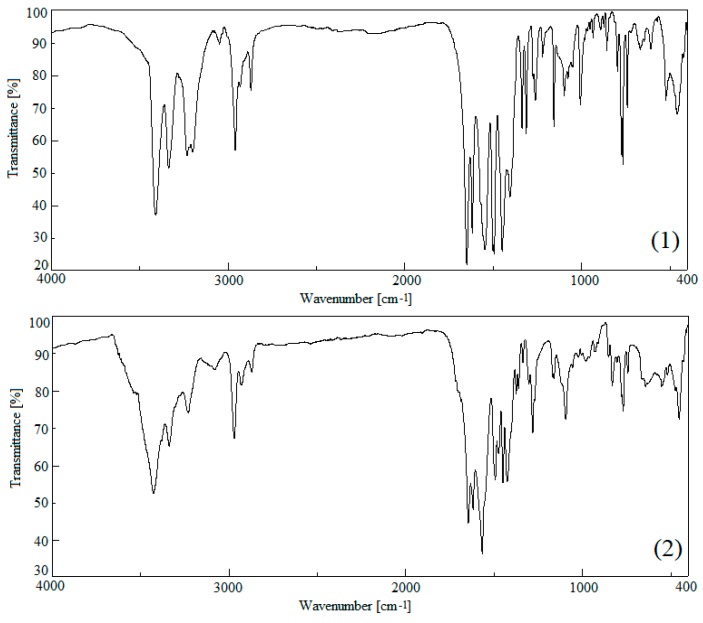
IR spectra of the studied compounds (**1** and **2**).

The IR spectra of compounds **1** and **2** contain bands characteristic for 2-apy and respective carboxylate anions (Table 5, Figure 6). The formation of a coordination bond by 2-apy through its aromatic nitrogen atom leads to stiffening of the aromatic ring. Consequently, energies of the most of CC, CN and CH oscillators of aromatic ring increase, what reflects in blue shifts of the corresponding bands in the spectra of coordination compounds, in comparison to the respective bands in the spectrum of pure 2-apy. Bands of asymmetric and symmetric stretching vibrations of the amino group are split into doublets in the spectra of studied compounds (both bands in **1** and the first one in **2**). This is a result of a presence of two 2-apy molecules in the coordination entity, and consequently, the existence of two different amino groups, of which each is involved in different intermolecular interactions, each amino group is involved in different intermolecular interactions. Moreover, two structurally different coordination entities exist in compound **1**, thus, the number of structurally different amino groups is 4. Comparing to pure 2-apy, the energy of *ν*_as_ NH_2_ and *ν*_s_ NH_2_ bands are also changed. In the studied compounds the first ones are red shifted while the second ones are blue shifted. The formation of more symmetrical hydrogen binding in the studied compounds (each hydrogen of each amino group is involved in one N—H•••O bond) than in pure 2-apy (only one hydrogen of amino group forms N—H•••N bond) [60] leads to the restraining of *ν*_s_ and the freeing of *ν*_as_. The strongest bands originating from vibrations within carboxylate anions correspond to asymmetric and symmetric stretching vibrations of a carboxylate group. A difference between wavenumbers of mentioned bands Δ*ν* (separation parameter [61,62]) is equal to 131 and 127 cm^−1^ respectively, for **1** and **2**. These values are in range characteristic for a bidentate chelating mode of carboxylate anions [23], thus it proves the usefulness of Δ*ν* to the determination of a binding mode of such anions.

### 3.4. TG Analysis

**Figure 7:**
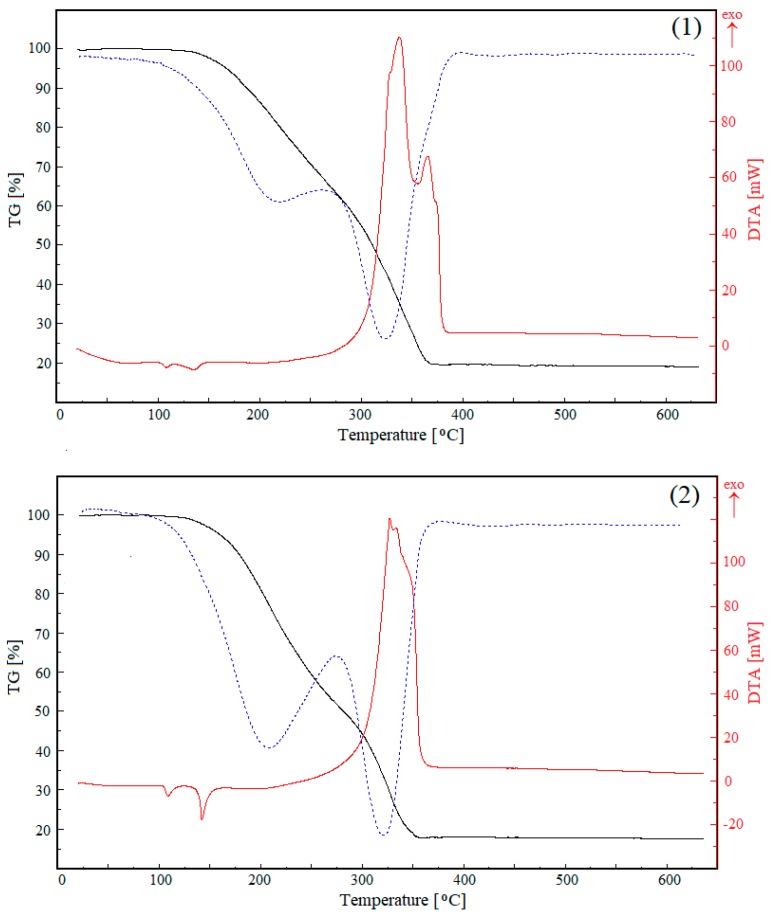
TG (black), DTG (blue dotted) and DTA (red) curves for the studied compounds (**1** and **2**).

Thermal decompositions of compounds **1** and **2** are one-step processes. DTG curves show two substages, but because of the processes overlapping, it is impossible to identify their exact temperature ranges (Figure 7). The compounds are stable up to 148 and 140 °C, respectively. Next, the simultaneous degradation of carboxylate anions and 2-apy molecules begins. The dominant volatile products determined by mass spectrometry are CH^+^, CO^+^, CO_2_^+^, NO^+^ and NO_2_^+^. Experimental mass losses (81.28% and 82.09%, respectively) are in agreement with theoretical ones (82.22%). The final product is cobalt(II) oxide, which is formed in a pure form above 367 and 358 °C respectively, for compounds **1** and **2**. Before the decomposition, two low energetic endothermic processes occur (at 108 and 142 °C for **1** and at 108 and 135 °C for **2**). The first one is connected with very small energy changes and can be associated with some structural changes within solids. The second one is a melting which is directly followed by the start of the decomposition.

### 3.5. Nanoparticles of Cobalt(II) Oxide

**Figure 8:**
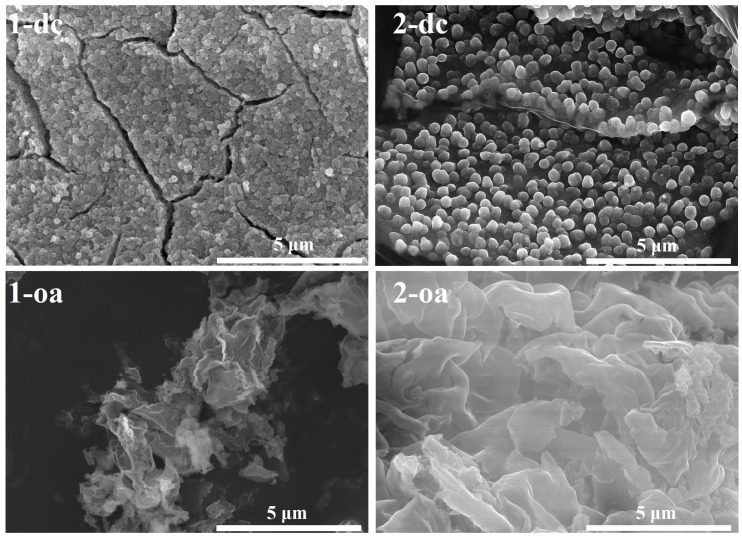
The SEM micrographs of CoO particles produced from **1** and **2** in two variants of the single precursor method (dc—direct conversion, oa—conversion with use of oleic acid).

Application of the studied compounds for fabrication of cobalt(II) oxide in two variants of the single precursors method leads to the formation of the most common cubic form of CoO (rock salt structure, a = b = c = 4.263 Å, Z = 4, space group Fm-3m). Isomorphous structures of coordination entities in both compounds cause that cobalt oxide particles produced in the same experiments possess similar habits (Figure 8). Direct conversion of compounds **1** and **2** allows formation of CoO spherical particles with average size 130 ± 40 nm and 350 ± 20 nm, respectively. The latter is characterized by the exceptionally narrow size distribution. On the other hand, CoO fabricated via conversion with the use of oleic acid adopts the form of irregular undulating sheets. In both synthesis variants, larger forms of CoO are produced by the use of **2**. It is a consequence of slightly closer packing of coordination moieties in **2** than in **1** (the respective molecular volumes are 501 and 510 Å^3^, and the difference is caused by shorter aliphatic chains of isobutyrate than of n-butyrate) which allows the formation of larger particles of CoO from compound **2**, i.e., larger distances between neighboring Co atoms of **1** cause larger contraction of them during the formation of CoO, and subsequently more frequent separation of the subsequently formed particles. The spherical shape is a typical morphology for CoO nanoparticles fabricated via direct conversion of coordination precursors. Similar nanoparticles were produced from i.a. [CoCl_2_(pyrazine)_2_]_n_ at 600 °C [65]. In case of a method with the use of dispersing liquid, polyhedral nanoparticles with well-shaped edges were synthesized the most frequently. The conversions of [Co(NCS)_2_(pyrazole)_4_] in oleic acid and (Co(acetylacetonate)_3_) in oleyamine, in comparable conditions to those applied in the current work, led to the formation of cuboidal and pyramidal CoO nanoparticles, respectively [66,67]. Therefore, the fabrication of CoO in the form of sheets is an untypical phenomenon. 

## 4. Conclusions

The two synthesized compounds containing hexacoordinated cobalt(II) cation possess similar molecular structures but distinctly differ in crystal packing. This effect originates from a small difference in one of the ligands, i.e., presence of n-butyrate or isobutyrate ions. Apart from the crystal packing, the spectral properties of both compounds also show some essential dissimilarities as a result of the presence of different isomers of the carboxylate anion, and subsequently different electronic transitions in the studied compounds. The study of the formation of nanoparticles in a single precursor method proved that even very small changes of molecular structure (in the presented case the isomerism of a small ligand) with retaining the same composition and coordination geometry, allow considerable alteration of the formed particles size (130 nm versus 350 nm). Such an approach is novel because typically the substantial changes of nano- and micro-particles size was attained by drastic changes of parameters of conversion used in the single precursor method, or total alteration of the structure of the precursor.

## Figures and Tables

**Table 1:** Crystal data and structure refinement details for the studied compounds.

Compound	1	2
Empirical formula	C_18_H_26_CoN_4_O_4_	C_18_H_26_CoN_4_O_4_
Formula weight	421.36	421.36
Crystal system	Orthorhombic	Orthorhombic
Space group	*Pna*2_1_ (No. 33)	*Pbca* (No. 61)
Temperature (K)	100.0(1)	100.0(1)
Wavelength (Å)	*λ*(Cu*Kα*) 1.54178	*λ*(Cu*Kα*) 1.54184
Unit cell dimensions (Å, °)	a = 15.3990(7)	a = 8.43060(10)
	b = 13.9101(6)	b = 21.4947(4)
	c = 19.0482(8)	c = 22.1522(6)
	α = 90.00	α = 90.00
	β = 90.00	β = 90.00
	γ = 90.00	γ = 90.00
Volume (Å^3^)	4080.2(3)	4014.27(14)
Z	8	8
Calculated density (Mg/m^3^)	1.35	1.37
Absorption coefficient (mm^−1^)	6.851	6.964
*F(000)*	1768	1768
Crystal size (mm)	0.095 × 0.107 × 0.119	0.019 × 0.072 × 0.099
*θ* Range for data collection (°)	2.869 to 72.385	3.991 to 80.219
Index ranges	−18 ≤ h ≤ 19, −17 ≤ k ≤ 17, −23 ≤ l ≤ 23	−10 ≤ h ≤ 10, −27 ≤ k ≤ 25, −28 ≤ l ≤ 28
Reflections collected/unique (R*_int_*)	45,837/7928 (0.0333)	72,898/4343 (0.0561)
Completeness to θ = 67° (%)	100.0	100.0
Min. and max. transmission	0.60175 and 1.00000	0.51741 and 1.00000
Data/restraints/parameters	7928/1/516	4343/0/260
Goodness-of-fit on *F*^2^	1.048	1.089
Final *R* indices (*I* > 2σ(*I*))	*R*1 = 0.0237, *wR*2 = 0.0602	*R*1 = 0.0303, *wR*2 = 0.0755
R indices (all data)	*R*1 = 0.0243, *wR*2 = 0.0606	*R*1 = 0.0364, *wR*2 = 0.0784
Largest diff. peak and hole (e•Å^−3^)	0.251 and −0.419	0.303 and −0.291

**Table 2:** Selected structural data of the studied compounds.

i—j	d_ij_ (Å)	*ν*_ij_ (v.u.)	i—j—k	α_ijk_ (°)	i—j—k	α_ijk_ (°)
Compound **1**						
Co1—O1	2.0749(16)	0.349	O1—Co1—O2	60.78(6)	O2—Co1—N3	88.38(7)
Co1—O2	2.2447(16)	0.220	O1—Co1—O3	95.06(7)	O3—Co1—O4	59.12(6)
Co1—O3	2.3271(19)	0.176	O1—Co1—O4	148.72(7)	O3—Co1—N1	93.03(7)
Co1—O4	2.0840(18)	0.340	O1—Co1—N1	97.59(7)	O3—Co1—N3	157.36(7)
Co1—N1	2.0758(18)	0.390	O1—Co1—N3	101.22(8)	O4—Co1—N1	100.88(7)
Co1—N3	2.110(2)	0.355	O2—Co1—O3	85.93(7)	O4—Co1—N3	100.06(7)
			O2—Co1—O4	97.35(7)	N1—Co1—N3	100.23(8)
			O2—Co1—N1	158.07(7)		
						
Co21—O21	2.6003(18)	0.084	O21—Co21—O22	55.36(7)	O22—Co21—N23	103.86(7)
Co21—O22	2.0110(19)	0.414	O21—Co21—O23	97.79(8)	O23—Co21—O24	60.29(6)
Co21—O23	2.0429(17)	0.380	O21—Co21—O24	89.79(8)	O23—Co21—N21	100.04(8)
Co21—O24	2.2833(16)	0.198	O21—Co21—N21	156.67(8)	O23—Co21—N23	97.53(7)
Co21—N21	2.106(2)	0.359	O21—Co21—N23	87.35(7)	O24—Co21—N21	86.11(7)
Co21—N23	2.0709(18)	0.395	O22—Co21—O23	143.99(8)	O24—Co21—N23	157.02(7)
			O22—Co21—O24	93.11(7)	N21—Co21—N23	105.01(8)
			O22—Co21—N21	101.90(7)		
Compound **2**						
Co1—O1	2.0847(11)	0.340	O1—Co1—O2	60.68(4)	O2—Co1—N3	164.60(5)
Co1—O2	2.2180(12)	0.237	O1—Co1—O3	147.00(5)	O3—Co1—O4	59.68(4)
Co1—O3	2.0765(11)	0.347	O1—Co1—O4	95.78(4)	O3—Co1—N1	105.14(5)
Co1—O4	2.3022(12)	0.189	O1—Co1—N1	99.57(5)	O3—Co1—N3	95.77(5)
Co1—N1	2.1010(13)	0.364	O1—Co1—N3	103.96(5)	O4—Co1—N1	164.42(5)
Co1—N3	2.0897(14)	0.375	O2—Co1—O3	97.49(5)	O4—Co1—N3	84.61(5)
			O2—Co1—O4	95.38(4)	N1—Co1—N3	94.18(5)
			O2—Co1—N1	89.88(5)		

**Table 3:** Hydrogen bonds and the first level graph motifs in the studied compounds (Å, °).

D–H•••A	d(D—H)	d(H•••A)	d(D•••A)	<(DHA)	G_d_^a^(n)
compound **1**					
N2—H2N•••O4	0.84(3)	2.35(3)	3.067(3)	143(3)	S(6)
N2—H2O•••O2 ^i^	0.81(3)	2.06(3)	2.861(3)	170(3)	C(6)
N4—H4N•••O21	0.81(4)	2.17(4)	2.973(3)	172(3)	D
N4—H4O•••O4	0.94(4)	1.93(4)	2.800(3)	153(3)	S(6)
N22—H22N•••O3 ^ii^	0.82(4)	2.08(4)	2.902(3)	175(3)	D
N22—H22O•••O22	0.73(4)	2.10(4)	2.799(3)	161(4)	S(6)
N24—H24N•••O22	0.84(4)	2.30(4)	3.054(3)	151(3)	S(6)
N24—H24O•••O24 ^iii^	0.84(3)	1.99(3)	2.822(3)	171(3)	C(6)
compound **2**					
N2—H2N•••O3	0.82(2)	2.10(2)	2.897(2)	164(2)	S(6)
N2—H2O•••O2 ^iv^	0.85(2)	2.04(2)	2.8733(19)	166(2)	C(6)
N4—H4N•••O4 ^v^	0.85(2)	2.12(2)	2.9546(19)	166.0(19)	R_2_^2^(12)
N4—H4O•••O1	0.86(2)	2.11(2)	2.9341(19)	161.0(19)	S(6)

Symmetry transformations used to generate equivalent atoms: ^i^ x + 0.5, −y + 1.5, z; ^ii^ x, y − 1, z; ^iii^ x + 0.5, −y + 0.5, z; ^iv^ x + 0.5, y, −z + 0.5; ^v^ −x + 1, −y + 1, −z + 1.

**Table 4:** The most important electronic transitions. H letter indicates HOMO, L – LUMO, α – α orbitals, β – β orbitals and +/-(number) represents subsequent orbitals above HOMO and LUMO, respectively.

Theoretical λ (nm)		E (eV)	*f*	The Most Important Orbitals Involved in Electronic Transitions	Character of Transition	Experimental λ (nm) (Solid State)		
1	2					1	2	2-apy
	220.76	5.6162	0.0349	αH-8 → αL + 1 βH → βL + 10	d(Co)/n(2-apy)/π(2-apy) → π*(2-apy)	217	215	
					d(Co)/n(ibut)/n(2-apy) → n(2-apy)			
223.29		5.5525	0.0238	βH-2 → βL + 7 αH-3 → αL + 5	d(Co)/n(2-apy)/π(2-apy) → d(Co)/n(2-apy)/π*(2-apy)			
					π(2-apy) → π*(2-apy)			
225.00		5.5104	0.0407	βH-2 → βL + 6	d(Co)/n(2-apy)/π(2-apy) → d(Co)/n(2-apy)			
	225.30	5.5030	0.0224	βH-7 → βL + 2 βH-7 → βL + 1 αH-3 → αL + 6	n(ibut) → d(co)/π*(2-apy)			
					n(ibut) → d(co)/π*(2-apy)			
					π(2-apy) → n(2-apy)			
246.94		5.0209	0.0077	βH-2 → βL + 3 βH-1→ βL + 5	d(Co)/n(2-apy)/π(2-apy) → d(Co)/n(2-apy)/π*(2-apy)	245	243	234
					d(Co)/n(2-apy)/n(but)/π(2-apy) → d(Co)/π*(2-apy)			
	249.10	4.9772	0.0051	βH → βL + 5	d(Co)/n(ibut)/n(2-apy) → d(Co)/π*(2-apy)			
	253.45	4.8918	0.0056	βH-4 → βL + 1 βH-4 → βL + 2	n(ibut) → d(co)/π*(2-apy)			
					n(ibut) → d(co)/π*(2-apy)			
259.67		4.7747	0.0062	βH-5 → βL + 1 βH → βL + 3	n(but) → π*(2-apy)			
					d(Co)/n(2-apy)/n(but) → d(Co)/n(2-apy)/π*(2-apy)			
263.31		4.7088	0.0105	βH → βL + 4 βH → βL + 3	d(Co)/n(2-apy)/n(but) → n(2-apy)/π*(2-apy)			
					d(Co)/n(2-apy)/n(but) → d(Co)/n(2-apy)/π*(2-apy)			
270.86		4.5775	0.0310	βH-2 → βL + 2 αH-2 → αL + 1 βH-7 → βL	d(Co)/n(2-apy)/π(2-apy) → d(Co)/π*(2-apy)	297	290	298
					π(2-apy)/n(2-apy) → π*(2-apy)			
					n(but) → d(Co)/π*(2-apy)			
276.20		4.4889	0.0389	βH-3 → βL + 1 αH-3 → αL	d(Co)/n(2-apy)/π(2-apy) → π*(2-apy)			
					π(2-apy) → π*(2-apy)			
	278.62	4.4500	0.0551	βH-2 → βL + 1 αH-2 → αL	d(Co)/π(2-apy) → d(Co)/π*(2-apy)			
					π(2-apy) → π*(2-apy)			
	305.73	4.0554	0.0149	βH-2 → βL	d(Co)/π(2-apy) → d(Co)/π*(2-apy)			
	314.31	3.9447	0.0070	βH → βL	d(Co)/n(ibut)/n(2-apy) → d(Co)/π*(2-apy)	329	325	
318.29		3.7702	0.0025	βH-4 → βL	n(but) → d(Co)/π*(2-apy)			
	316.39	3.9187	0.0065	αH → αL	d(Co)/n(ibut)/n(2-apy) → π*(2-apy)			
	326.13	3.8017	0.0025	βH → βL + 1 αH-3 → αL + 4	d(Co)/n(ibut)/n(2-apy) → d(Co)/π*(2-apy)			
					π(2-apy) → π*(2-apy)			
	329.02	3.7683	0.0021	αH-2 → αL + 3	π(2-apy) → π*(2-apy)			
333.03		3.7229	0.0067	βH → βL + 1	d(Co)/n(2-apy)/n(but) → π*(2-apy)			
361.20		3.4325	0.0002	αH-3 → αL βH-3 → βL + 1	π(2-apy) → π*(2-apy)	373	367	
					d(Co)/n(2-apy)/π(2-apy) → d(Co)/π*(2-apy)			
	367.12	3.3772	0.0001	αH-3 → αL + 1 βH-3 → βL + 1	π(2-apy) → π*(2-apy)			
					d(Co)π(2-apy) → d(Co)/π*(2-apy)			
368.90		3.3609	0.0003	αH-2 → αL + 1 βH-2 → βL	π(2-apy)/n(2-apy) → π*(2-apy)			
					d(Co)/n(2-apy)/π(2-apy) → d(Co)/π*(2-apy)			
	378.50	3.2757	00030	αH-2 → αL	π(2-apy) → π*(2-apy)			
				βH-2 → βL	d(Co)/n(2-apy)/π(2-apy) → d(Co)/π*(2-apy)			
	537.58	2.3063	0.0006	βH-1 → βL + 9 βH-1 → βL + 7	d(Co)/n(ibut)π(2-apy) → d(Co)/n(ibut)/n(2-apy)	485	492	
					d(Co)/n(ibut)π(2-apy) → d(Co)/n(ibut)/n(2-apy)/π*(2-apy)			
539.69		2.2973	0.0013	βH-1 → βL + 9	d(Co)/n(2-apy)/n(but)/π(2-apy) → d(Co)/n(2-apy)			
	591.11	2.0975	0.0013	βH → βL + 7	d(Co)//n(ibut)n(2-apy) → d(Co)/n(ibut)/n(2-apy)/π*(2-apy)	532	521	
604.34		2.0516	0.0015	βH-1 → βL + 9 βH → βL + 8	d(Co)/n(2-apy)/n(but)/π(2-apy) → d(Co)/n(2-apy)			
					d(Co)/n(2-apy)/n(but) → d(Co)/π*(2-apy)			

Used abbreviations: d(Co)—d orbital of cobalt cation, n(but)—non-bonding orbital of butyrate anion, n(ibut)—non-bonding orbital of isobutyrate anion, n(2-apy)—non-bonding orbital of 2-aminopyridine, π(2-apy)—π orbital of 2-aminopyridine, π*(2-apy)—π antibonding orbital of 2-aminopyridine.

**Table 5:** Vibrational frequencies (cm^−1^) and their assignments.

1	Cobalt Butyrate [23,38]	2-apy [63,64]	Assignment	2	Cobalt Isobutyrate [23,38]	2-apy [63,64]	Assignment
3410 s		3446 s	*ν*_as_ NH_2_	3430 s		3446 s	*ν*_as_ NH_2_
3338 m			*ν*_as_ NH_2_	3343 m			*ν*_as_ NH_2_
3233 m			*ν*_s_ NH_2_	3230 m		3188 m	*ν*_s_ NH_2_
3201 m		3188 m	*ν*_s_ NH_2_				
3052 w		3026 w	*ν* CH(ar)	3078 w		3026 w	*ν* CH(ar)
2960 m	2962 s		*ν*_as_ CH_3_	2970 m	2967 m		*ν*_as_ CH_3_
2934 w	2936 w		*ν*_s_ CH_3_	2928 w	2929 w		*ν*_s_ CH_3_
2870 m	2876 m		*ν*_s_ CH_2_	2870 w	2872 w		*ν* CH
1648 s		1628 s	*σ* NH_2_	1646 s		1628 s	*σ* NH_2_
1617 s		1605 m	*σ* NH_2_, *ν* CC(ar)	1617 s		1605 m	*σ* NH_2_, *ν* CC(ar)
1547 s	1557 s		*ν*_as_ COO	1552 s	1557 s		*ν*_as_ COO
1494 s		1488 s	*δ-α* CHar	1492 m		1488 s	*δ-α* CH(ar)
				1476 m	1472 m		*δ*_as_ CH_3_
1449 s	1448 m	1440 s	*δ*_as_ CH_3_, *δ-α* CH(ar)	1450 m		1440 s	*δ-α* CH(ar)
1416 m	1408 s		*ν*_s_ COO	1425 m	1416 s		*ν*_s_ COO
				1375 w	1376 w		*δ*_s_ CH_3_
				1361 w	1361 m		*δ*_s_ CH_3_
1336 m	1332 m	1340 m	*δ*_s_ CH_3_, *δ-α* CH(ar)	1337 w		1340 m	*δ-α* CH(ar)
1312 m	1314 m	1324 m	*ω* CH_2_, *ν* CN(NH_2_)				
				1303 w	1307 m		*δ* C_α_H
1260 m	1245 m	1275 m	*τ* CH_2,_ *ν* CN(ar)	1281 m	1284 m	1275 m	*δ* C_α_H, *ν* CN(ar)
1220 w	1212 w		*τ* CH_2_				
				1167 w	1168 m		*ρ*_as_ CH_3_
1155 m		1156 m	*δ-α* CH(ar)	1159 w		1156 m	*δ-α* CH(ar)
1098 w	1100 w		*ν* C_β_C_α_	1094 m	1098 m		*ρ*_s_ CH_3_
1076 w	1079 w		*δ*-*γ* CH_3_				
1049 w	1047 w	1044 w	*δ*-*γ* CH_3_, *ν* CN(ar), *ν* CC(ar)	1054 w		1044 w	*ν* CN(ar), *ν* CC(ar)
1006 m	1015 w	987 m	*δ*-*γ* CH_3_, *δ*-*γ* CH(ar)	980 w		987 m	*δ*-*γ* CH(ar)
934 w	934 m		*ν* C_γ_C_β_	930 w	925 w		*ρ*_s_ CH_3_
890 vw	893 m		*ν* C_α_C				
872 vw	879 w		*ν* C_α_C				
857 w		860 w	*δ*-*γ* CH(ar)	850 w		860 w	*δ*-*γ* CH(ar)
				831 m	845 w		*ν*_s_ (C_β_)_2_C_α_C, *σ* COO
796 m	800 m		*δ*-*γ* CH_2_				
766 s	753 m	772 s	*δ*-*γ* CH_2_, *T* ar	768 m	762 w	772 s	*ω* COO*, T* ar
741 m	720 m	736 w	*δ*-*γ* CH_2_, *T* ar	742 w		736 w	*T* ar
664 vw	645 w	665 m	*σ* COO, *δ-α* ar	641 w	664 w	665 m	*σ* COO, *δ-α* ar
607 w	585 w		*ω* COO				
				552 w	560 w		*δ*C_α_C
522 w		522 s	*T* ar	518 w		522 s	*T* ar
454 m		435 m	*ω* NH_2_	451 m		435 m	*ω* NH_2_

Vibrations symbols: w—weak, m—medium, s—strong, *ν*—stretching, *δ*—bending, *σ*—scissoring, *ρ*—rocking, *τ*—twisting, *ω*—wagging, *T—*torsional, *α*—in plane, *γ*—out of plane, as—asymmetric, s—symmetric, ar—aromatic ring.

## Supplementary Materials

The following are available online at https://www.mdpi.com/1420-3049/24/18/3357/s1, Figures S1, S2, S3, and S4 are available online in the supplementary materials.
