# Supplementary material for: Structural Insights into Influence of Isomerism on Properties of Open Shell Cobalt Coordination System
Source: Molecules. 2019 Sep 16;24(18):3357. doi: 10.3390/molecules24183357 (PMC6767358; doi:10.3390/molecules24183357)
Supplement: Supplementary file 1 [file molecules-24-03357-s001.pdf]

Supplementary materials

Structural Insights into Influence of Isomerism on Properties of Open Shell Cobalt Coordination System

Marcin Swiatkowski, Tomasz Sieranski, Marta Bogdan and Rafal Kruszynski *

Institute of General and Ecological Chemistry, Lodz University of Technology, Zeromskiego 116, 90-924 Lodz, Poland; marcin.swiatkowski@p.lodz.pl (M.S.); tomasz.sieranski@p.lodz.pl (T.S.); 801084@edu.p.lodz.pl (M.B.)

* Correspondence: rafal.kruszynski@p.lodz.pl

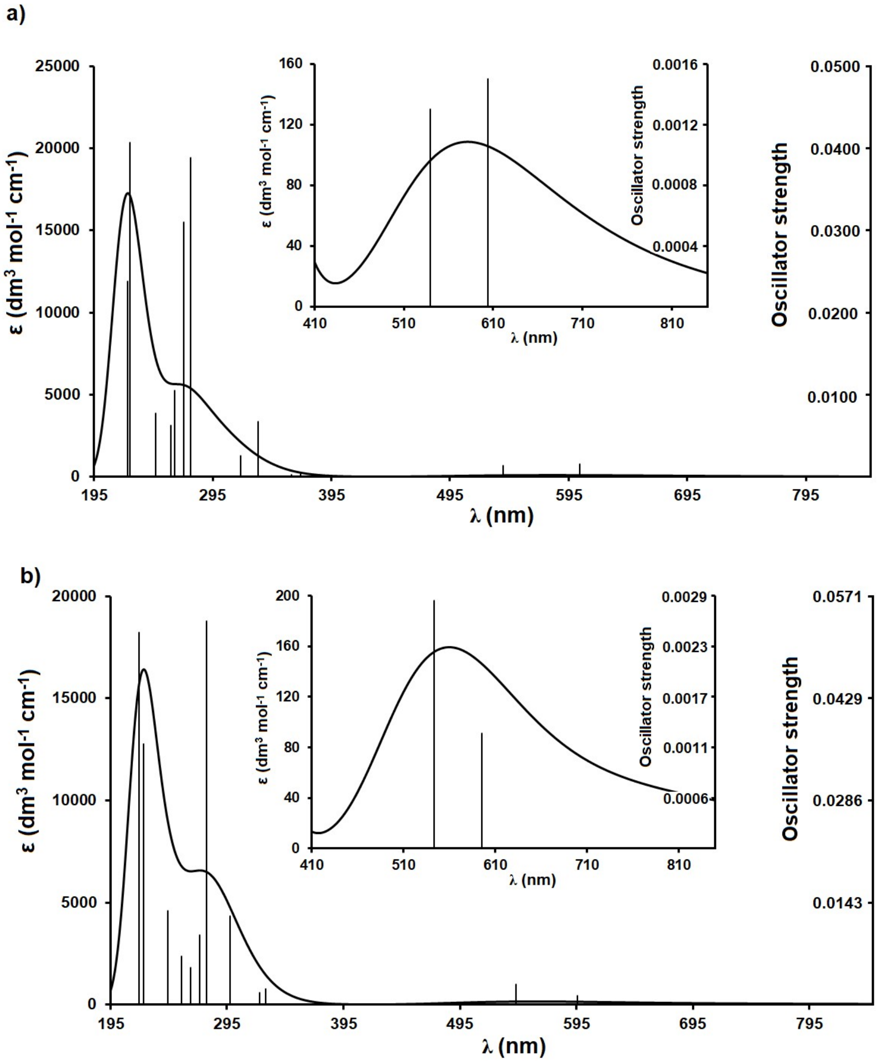

Figure S1. Calculated UV-Vis spectra for both coordination units of **1**: a) containing Co1 atom, b) containing Co21 atom . The most important oscillator strengths are shown as vertical black lines.

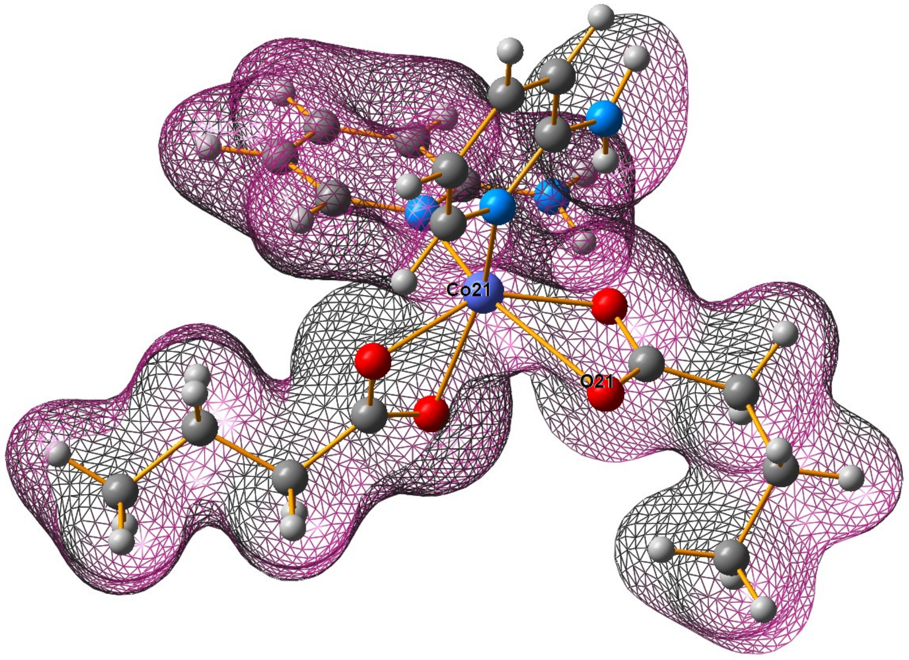

Figure S2. Isodensity of total density surface of **1** (with 0.015 au) showing the electron density along the axis of Co21-O21 bond. The total density was calculated at B3LYP/6-31++g(2d,2p) level of theory. For clarity, the frontmost portions of the surface has been partially removed.

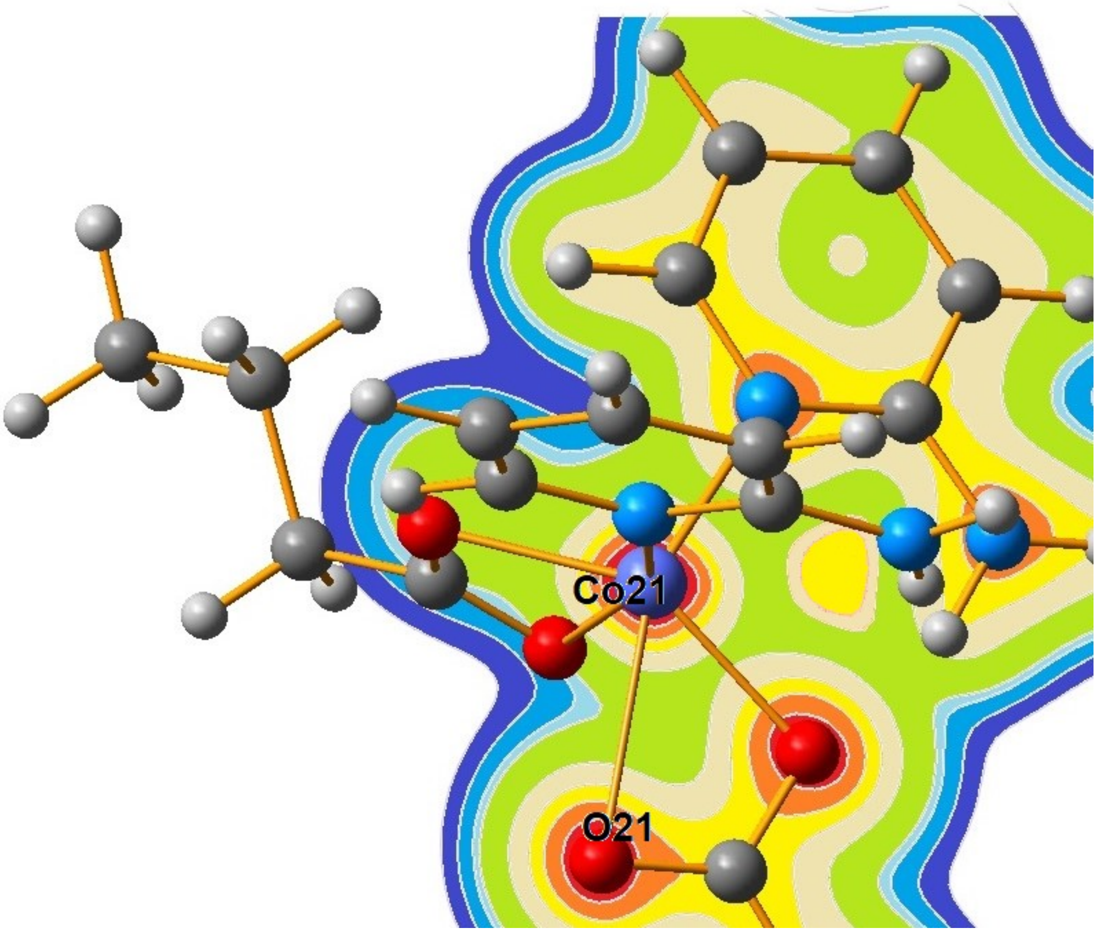

Figure S3. The calculated charge density in Co21/O21O22 plane of compound **1**. The total density was calculated at B3LYP/6-31++g(2d,2p) level of theory.

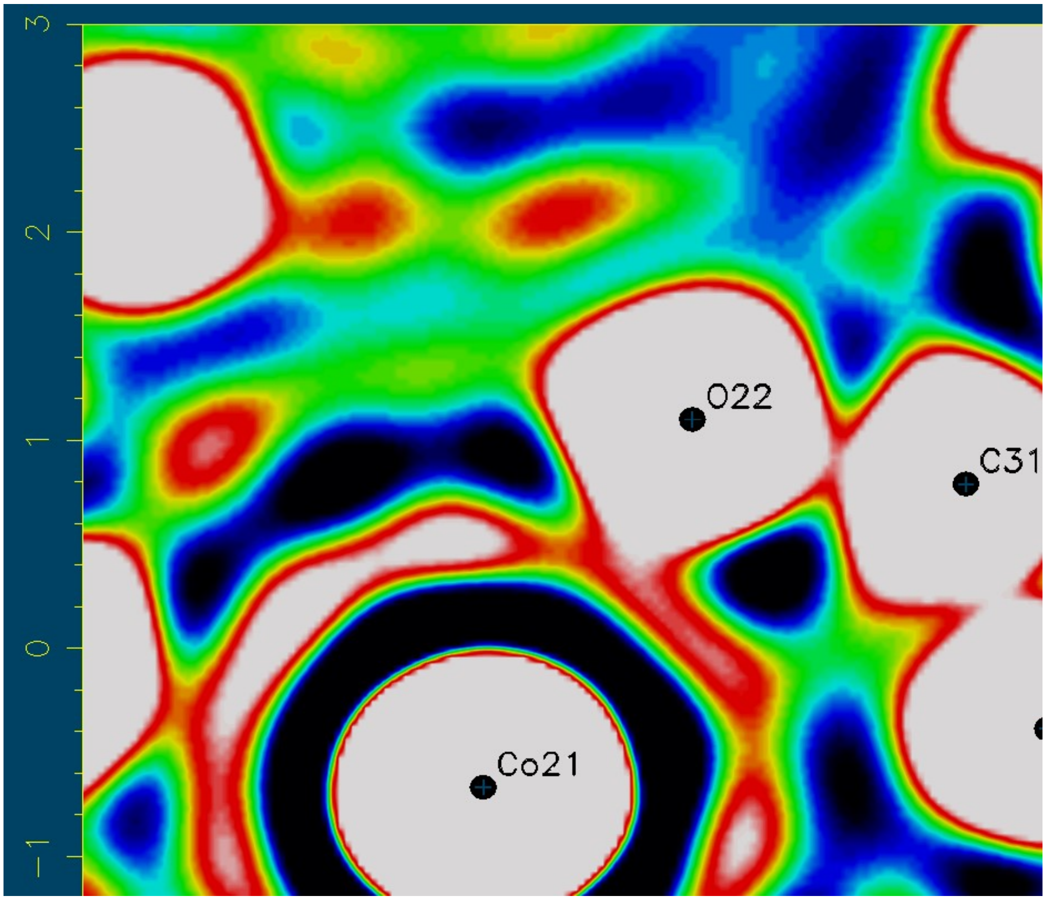

Figure S4. The measured electron density (F_o) in Co21/O21O22 plane of compound **1**. The F_o was generated from the observed diffraction intensities with WinGX [L. J. Farrugia, J. Appl. Crystallogr., 1999, 32, 837-838.].
